# Spinal Cord Injury Management through the Combination of Stem Cells and Implantable 3D Bioprinted Platforms

**DOI:** 10.3390/cells10113189

**Published:** 2021-11-16

**Authors:** Atefeh Zarepour, Sara Hooshmand, Aylin Gökmen, Ali Zarrabi, Ebrahim Mostafavi

**Affiliations:** 1 Department of Biomedical Engineering, Faculty of Engineering and Natural Sciences, Istinye University, Istanbul 34396, Turkey; atefeh.zarepour@gmail.com; 2 Nanotechnology Research and Application Center (SUNUM), Sabanci University, Istanbul 34956, Turkey; sara.houshmand@sabanciuniv.edu; 3 Molecular Biology and Genetics Department, Faculty of Engineering and Natural Sciences, Bahcesehir University, Istanbul 34353, Turkey; aylingokmen00@gmail.com; 4 Stanford Cardiovascular Institute, Stanford University School of Medicine, Stanford, CA 94305, USA; 5 Department of Medicine, Stanford University School of Medicine, Stanford, CA 94305, USA

**Keywords:** spinal cord injury, stem cells, 3D bioprinting, tissue regeneration, neural tissue engineering

## Abstract

Spinal cord injury (SCI) has a major impact on affected patients due to its pathological consequences and absence of capacity for self-repair. Currently available therapies are unable to restore lost neural functions. Thus, there is a pressing need to develop novel treatments that will promote functional repair after SCI. Several experimental approaches have been explored to tackle SCI, including the combination of stem cells and 3D bioprinting. Implanted multipotent stem cells with self-renewing capacity and the ability to differentiate to a diversity of cell types are promising candidates for replacing dead cells in injured sites and restoring disrupted neural circuits. However, implanted stem cells need protection from the inflammatory agents in the injured area and support to guide them to appropriate differentiation. Not only are 3D bioprinted scaffolds able to protect stem cells, but they can also promote their differentiation and functional integration at the site of injury. In this review, we showcase some recent advances in the use of stem cells for the treatment of SCI, different types of 3D bioprinting methods, and the combined application of stem cells and 3D bioprinting technique for effective repair of SCI.

## 1. Introduction

Spinal cord injury (SCI) results in irreversible loss of sensory, motor, and autonomic functions [1]. Close to 500,000 individuals suffer from SCI every year (https://sciprogress.com/understanding-spinal-cord-injuries/ (accessed on 10 December 2020)). SCI affects not only patients’ body function but also their socioeconomic conditions, and the disorder is often associated with considerable individual suffering and societal costs [2]. Current therapeutic options, which aim to minimize the extent of secondary damage and support recovery of function through rehabilitative measures [3,4], are inadequate. Therefore, it is critical to develop an effective treatment for SCI.

Researchers have attempted to identify new treatments, such as drug administration, surgical decompression, hypothermia, and stem cells therapies, which could promote nerve cell regeneration, repair damaged parts, reduce the metabolic rate, and suppress acute inflammatory processes (to protect central nerve tissue from continued injury following traumatic damage) [5,6]. Recently, new therapeutic methods, such as cell/gene therapy, motor exercises, electrical stimulation, electrochemical neuromodulation therapy, and stem cell-based therapies, have been used, alone or in a combinatory approach, to achieve successful SCI therapy [7].

Stem cell-based therapies include applications with high potential, particularly in the SCI field, because of their self-renewable properties. Transplantation of stem cells is considered a promising strategy for the regeneration of dead cells or lost tissue caused by SCI, especially when combined with particular three-dimensional (3D) printed materials/scaffolds. Three-dimensional printing is a type of additive manufacturing (AM) developed by Charles Hull in 1986 based on the idea of “utilizing layers of materials on top of each other for printing a subject”. Nowadays, 3D printing technology is applied in different fields and is especially used for biomedical applications. This has led to the emergence of a new type of 3D printing technology, known as 3D bioprinting, which is used for the creation of a cell pattern that could preserve the viability and functionality of cells [8,9].

The combinatory approach of using 3D printed scaffolds and stem cells could be more effective in improving motor function in SCI than using a single therapeutic method [10]. Additionally, this combined therapy could enhance cell proliferation and neural differentiation in vitro while reducing inflammation and the formation of a cavity in vivo. This method shows an anti-inflammatory effect via suppressing the activated microglia or macrophages in the injury site [11]. That is, implantable polymeric scaffolds could be implanted to fill the gap created by SCI in the injury site and rejuvenate the axons in the restorative environment [12]. In SCI repair, biomaterial scaffolds play a crucial role in generating a microenvironment favorable for regeneration [13]; for instance, in addition to playing the role of a supporting platform, these biomaterial scaffolds could be employed as carriers for seeded cells to repair the microenvironment at the SCI site and, finally, to bridge the deficiency in SCI [14]. They could be used in a 3D porous structure to provide a suitable surface for cell growth and proliferation, as well as supporting the flow of nutrients, oxygen, and discharge of metabolites [15]. Moreover, different types of biological components could be applied in the structure of these scaffolds, thereby stimulating stem cell–scaffold interactions and promoting their therapeutic performance [16].

The main focus of this overview is to introduce the combined therapy of stem cells and different types of 3D printed scaffolds for SCI therapy. In this review, we discuss the utilization of different types of stem cells used for SCI, their mechanism of function, and new types of 3D bioprinted scaffolds used as platforms for stem cells in SCI therapy. A combinational approach using implantable platforms and stem cell therapy could provide novel and promising strategies for future studies and show potential for safe applications in further clinical trials.

## 2. Stem Cell Therapy

As a considerable challenge, SCI therapy has always been of great concern among clinical scientists due to its consequent irreparable tissue damage and constant sensorimotor impairment [17]. Stem cells are the only raw cells that could provide a repair system in the body through their natural ability to produce any type of specialized cells. These are cell lines with self-renewing capability, which makes them suitable for the treatment of SCI [18,19]. However, the wide application of stem cells in this field is restricted by their limited sources and immune rejection, as well as the ethical issues related to the transplantation of some types of stem cells [20]. Thus, to encourage further advances in the transplantation of stem cells to manage SCI, it is critical to use effective stem cell lines to prevent ethical issues and immune rejection [21]. Microenvironmental changes that occur in the injury site of the spinal cord could subsequently stop axonal regeneration. Stem cells could provide axonal trophic support to these microenvironments and consequently enhance vascularization, modify the inflammatory responses, and prevent cystic changes. To design such beneficial microenvironments for spinal cord restoration, stem cell therapy has been combined with other therapeutic methods, such as gene therapy, in which therapeutic effects of specific genes are transferred to the site of injury. These stem cells can replace injured neural cells and upregulate specific neurotrophic factors to reconstruct the spinal cord neural circuit [22].

Different stem cells could also be combined and transplanted to perform more effectively than a single cell in repairing SCI. This combination of different cell types enables unique neurotrophic factors in the injured area to stimulate axonal regeneration. Despite considerable effort in the field, further investigations should be performed to obtain optimal combination parameters and accomplish the greatest functional recovery from SCI [23].

### 2.1. Therapeutic Mechanisms of Stem Cells

Stem cells can be used for spinal cord therapy, as they tissue repair, neurotrophy, angiogenesis regeneration, and promotion (reconstruction of neurovascular units) [24]. Transplanted stem cells show anti-apoptotic effects by disturbing the balance between anti-apoptotic and pro-apoptotic factors and, thus, improving the survival of the tissue and enhancing neurological function resumption. They also exhibit anti-inflammatory effects by reducing the number of neutrophils and the expression of inflammatory proteins that upgrade the neurological function [25].

In some cases, the efficiency of spinal cord treatment is depended on the type of transplanted cells. For example, neuronal stem cells accelerate the process of SCI treatment by direct release of neurotrophic factors, such as the glial fibrillary acidic protein (GFAP), B-cell lymphoma-2 (Bcl-2), neurotrophin-3 (NT-3), and brain-derived neurotrophic factor (BDNF) [26].

Several therapeutic approaches have been proposed for stem cell-based treatments of SCI [27]. Stem cell-based approaches could regenerate the neuronal circuit by replacing the spinal cord’s damaged neurons via the creation of new links with the host neurons in the spinal cord. Moreover, the formation of synapses and regeneration of axons could be promoted after stem cell transplantation via the interactions between stem cells and the surrounding tissues. This could also modify the injury site microenvironment and accelerate the growth of the neural axon by generating some neurotrophic growth factors [28]. Transplanted stem cells could also enhance the myelin formation around neural axons, both newly grown and previous ones, by differentiating into gliocytes and oligodendrocytes. This would lead to an increase in functional recovery of patients with spinal cord injuries, which is substantial in the repair process [29].

Stem cells could also be applied in combination with other therapeutic mechanisms that improve their effectiveness. For example, utilizing gene-modified stem cells, which express high levels of neurotrophic factors and neuronal cell adhesion molecules, could be a promising method for SCI treatment. It is noteworthy that this is a new therapeutic method, and more investigations are needed in this field to clarify all aspects in patients [30]. Nanomaterials could also be used as carriers of different types of drugs, delivering them to their targeted site without side effects on other parts, thus increasing the bioavailability of their cargos at the injury site. Based on the types of materials used for the fabrication of these nanomaterials, they could also affect the motor axon regeneration or inhibit inflammatory effects, which could help stem cells in proliferation and differentiation and promote SCI treatment [31].

Among the most promising tools that could improve the effectiveness of stem cells for spinal cord therapy are implantable scaffolds, especially 3D printed ones that are used at the site of injury. These multi-purpose tools fabricated from natural or synthetic agents could act as a niche for the transplantation of stem cells. They are fabricated from biocompatible materials and could directly affect the injured area by improving axonal regeneration [32].

### 2.2. Different Types of Stem Cells Used for SCI

Various types of stem cells have been applied to restore spinal cord injuries, including embryonic stem cells, induced pluripotent stem cells (iPSCs), mesenchymal stem cells, and neural stem cells [33]. Some of the most prevalent of these cells are described in detail in the following sections.

#### 2.2.1. Embryonic Stem Cells

Pluripotent embryonic stem cells are one of the interesting classes of stem cells derived from early-stage embryos. They show an unlimited dividing ability in a homogeneous state and the capability to convert into multiplex somatic cell types [34].

The efficiency of the transplantation of human embryonic stem cells as a promising approach in the treatment of SCI has been assessed via several studies. In a recent preclinical study, human embryonic stem cell-derived neural crest cells were tested as therapeutic candidates in an SCI model of adult rats. Utilizing these cells led to the enhancement of the sprouting function and partial recovery of the forelimb motor after being transplanted into both chronic and acute cervical SCI rat models. They could also stimulate the remodeling of descending raphespinal projections by producing different biologically active trophic factors [35].

In a recent survey, human embryonic stem cell-derived neural stem cells (hESC-NS) were transplanted into an SCI rat model via utilizing a type of hyaluronic acid-based hydrogel to evaluate the effect of cells on the regeneration of tissue and recovery of movement. According to the results of this study, the presence of hydrogel led to not only the differentiation of stem cells into three different types of neuronal cells (astrocytes, neurons, and oligodendrocytes) but also to enhancement of the neuronal myelination at the injury site. Moreover, the animal movements were improved after seven weeks of injury by developing locomotor functions [36].

#### 2.2.2. Induced Pluripotent Stem Cells (iPSC)

Due its various advantages, iPSCs technology has been applied as a promising alternative therapy for difficult-to-treat spinal cord injuries in escalating morbidity cases. iPSCs are artificial stem cells with proliferative and self-renewal abilities that are derived from somatic cells via their reprogramming through the expression of defined pluripotency-associated factors. These cells can easily be differentiated into the precursor cells of all neural cell types at the SCI site and demonstrate remarkable potential in SCI therapeutic applications [37,38].

The combined use of iPSC-derived neural stem cells and long non-coding RNA-growth arrest-specific transcript 5 (lncRNA-GAS5) could inhibit neuronal apoptosis and promote repair of the SCI. The results of a Western blot analysis indicated an increase in the expression level of B cell lymphoma/leukemia-2 along with a decrease in the amount of Bcl-2 associated X protein, cytochrome C, and cleaved caspase-3. Morphological observations confirmed that the transplantation of cells could improve renovation in mouse model of SCI, while the restorative effects of the transplantation were inhibited by the growth arrest-specific 5 (GAS5) silencing gene [39].

A critical point about utilizing iPSCs for SCI treatment is the regional identity of the neural progenitor cells (NPCs) derived from iPSCs. This factor was assessed in a study in which two types of NPCs (forebrain- and spinal cord-type NPCs) were fabricated from human iPSCs and transplanted into an SCI mouse model. The results of this study revealed that only mice treated with spinal cord-type NPCs showed an improvement in their motor recovery, and the other types of cells could not exhibit any significant effect on SCI treatment [40].

#### 2.2.3. Mesenchymal Stem Cells

Mesenchymal stem cells are the best candidates for the treatment of SCI, and they have been widely applied in regenerative medicine due to their unique characteristics in injured tissues, such as high availability, multi-potency, immunomodulation, and self-regeneration, as well as being easily isolated and cultured [41]. However, this modern medication has shown minimal progress in developing injured nerve functions in SCI patients, mainly due to the complicated pathophysiological variations that appear after the occurrence of injuries [22].

**Figure 1:**
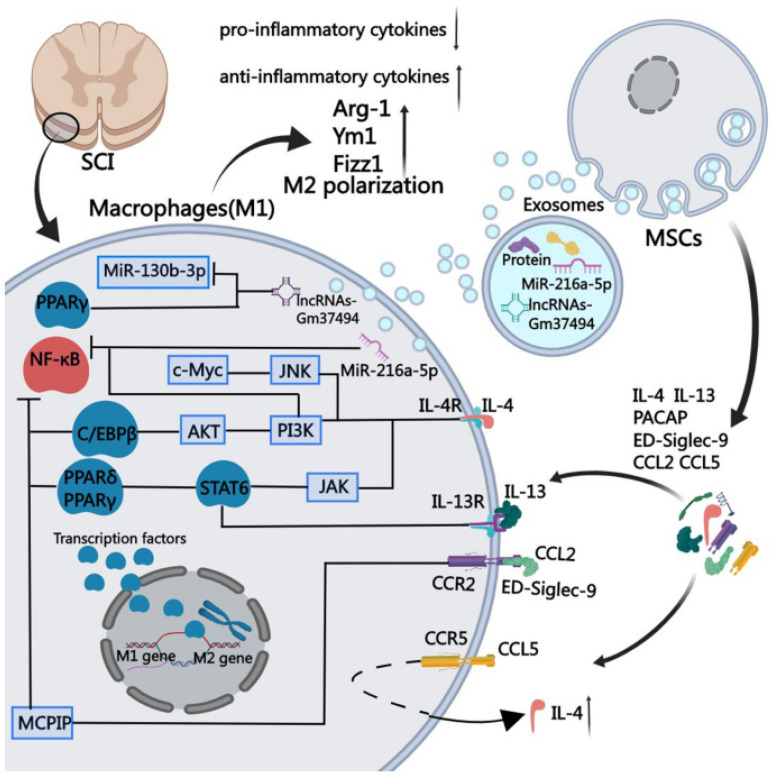
Mechanism of regulation of macrophage polarization by mesenchymal stem cells by stimulating them at the site of SCI, which could produce soluble proteins of chemokine (C-C motif) ligand 2 (CCL2), chemokine (C-C motif) ligand 5 (CCL5), interleukin 4 (IL-4), interleukin 13 (IL-13), sialic acid-binding Ig-like lectin 9 (ED-Siglec-9), and pituitary adenylate cyclase-activating peptide (PACAP) [42].

Mesenchymal stem cells could repair SCI through the macrophage polarization mechanism. The functions of immune cells at various SCI sites may differ over time and cause post-inflammatory reactions that could lead to secondary injuries at the injury site. The key mediators of inflammatory responses after SCI are two different subtypes of macrophages, classically activated macrophages (M1 macrophages) and the alternatively activated macrophages (M2 macrophages), or pro-inflammatory and anti-inflammatory macrophages, respectively, which have immunoregulatory potentials. That is, the activation of M1 macrophages is accompanied by elevated amounts of oxidative metabolites (such as nitric oxide and superoxide) and pro-inflammatory cytokines that could damage the healthy tissues around the injured region, while M2 macrophages could promote tissue regeneration and provide resistance against body inflammation. The therapeutic effect of mesenchymal stem cells is due to their particular immune microenvironments, which impact macrophage polarization and subsequently control the effects of secondary injury (after SCI) by establishing higher recovery of nerve functions (Figure 1) [42].

The implantation effects of mesenchymal stem cell progenitors on animals and clinical models of SCI have also been studied. These cells could be obtained from different tissues, such as adipose tissue, bone marrow, or Wharton jelly, easily expanded in vitro due to their multi-lineage differentiation potential and adjusted dendritic cell activity owing to their immunomodulatory and paracrine potential. Since they are hypoimmunogenic, they could result in better regeneration by transferring to lesion sites to protect perineuronal nets and promote neural plasticity [43].

#### 2.2.4. Neural Stem Cells (NSCs)

**Figure 2:**
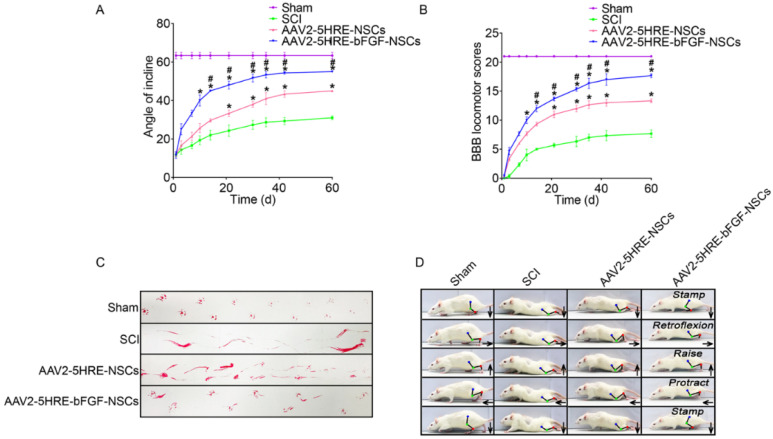

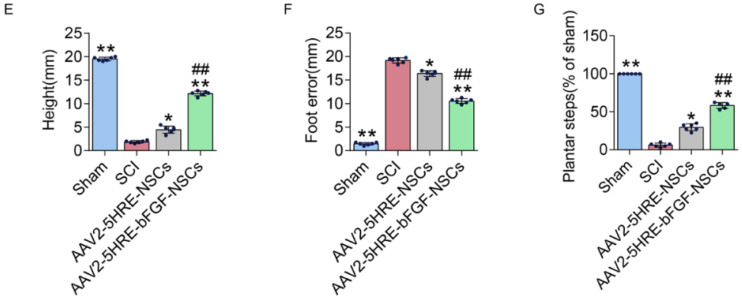
(**A**) The inclined plane test scores of sham, SCI group, adeno-associated virus 2-5HRE- neural stem cell (AAV2-5HRE-NSC) group, and AAV2-5HRE-basic FGF-NSC group. (**B**) The Basso–Beattie–Bresnahan (BBB) scales of sham, SCI group, AAV2-5HRE-NSC group, and AAV2-5HRE-bFGF-NSC group. Data are reported as the mean values  ±  SEM (*n*  =  3). (**C**) Footprint examinations of the four studied groups. (**D**) Video sequences of a rat walking 2 months after SCI. Arrows indicate foot movement. Scale bar =  20 mm. (**E**) Height of the trunk from the ground reported as weight support, (**F**) foot error, and (**G**) plantar step. Data are reported as the mean values  ±  SEM (*n*  =  6). # attributed to the *p*  <  0.05, AAV2-5HRE-NSCs group verse SCI group, * is attributed to the *p*  <  0.05, AAV2-5HRE-bFGF-NSCs verse AAV2-5HRE-NSCs group, ** is represented the *p*  <  0.01, versus the SCI group or sham group, and finally ## is represented the *p* <  0.01, versus the AAV2-5HRE-NSCs group [45].

Neural stem cells are another type of stem cells that are being progressively applied in SCI treatment. They are a multipotent subtype of progenitor cells in the nervous system and can be derived from three main sources: primary central nervous system (CNS) tissue, pluripotent stem cells, and somatic cells. Similar to other types of stem cells, they have a self-renewing ability and can easily differentiate into different types of neuronal cells, such as neurons, oligodendrocytes, and astrocytes [44]. After spinal cord injury, transplanted NSCs could play a key role in nerve regeneration and nutrition [26]. The combined use of NSCs and fibroblast growth factor (FGF) has revealed promising effects in the therapy of SCI; however, employing these strategies to repair SCI is still a challenging issue due to hostile hypoxia conditions. Indeed, hostile hypoxia conditions do not allow stem cells to survive and self-regulate, and it is necessary to provide a condition that could overcome these challenges and lead to regulation of the expression of FGF and an improvement in cell survival. In a recent study, a prototype adeno-associated virus (AAV2) was transduced into NSCs that can regulate the expression of the FGF gene under the hypoxia response element (HRE) condition (AAV2-5HRE-bFGF-NSCs). According to a mechanistic study, this therapy could increase the expression of basic FGF and enhance total functional recovery compared to the control group (AAV2-5HRE-NSCs) by increasing the expression of specific neuronal proteins and reducing the expression of the glial fibrillary acidic protein and autophagy-associated proteins (Figure 2). Overall, AAV2-5HRE-bFGF-NSCs treatment could upgrade the recovery of SCI in rats by enhancing successful nerve regeneration as well as inhibiting cell autophagy and glial scar formation [45].

Genetically modified human NSCs (hNSCs) were applied to detect the stimulating effect of these cells on the functional recovery of the injured spinal cord. The results of MRI tests proved the migration of transplanted cells toward the injury site of the spinal cord that could functionally connect to host neurons and substantially enhance locomotor functions while expressing neural lineage markers. These results imply that the transplantation of these cells could regulate inflammatory cells and glia activation and improve the hyperalgesia that occurred after SCI [45].

Moreover, the combination transplantation of NSCs and human olfactory ensheathing mucosa cells has shown effectiveness in preclinical trials to treat post-traumatic cysts of spinal cord injuries. This co-transplantation improved the hind limbs’ motor activity and led to a cyst size reduction of 4–12% in rats [46]. Some of the advantages and limitations of different types of stem cells are summarized in Table 1.

Due to the critical role of biomaterial scaffolds in forming a beneficial regeneration microenvironment for repairing injured spinal cord tissues, they have been used as supporting and carrier platforms for stem cells. The combined use of stem cells and scaffolds could facilitate the reconstruction of the microenvironment at the injury site [50]. Moreover, immobilizing cells into scaffolds could control the diffusion rate after implantation [15]. In the following section, recent developments in the preparation of 3D printed scaffolds and their combined use with different types of stem cells for SCI treatments are reviewed.

## 3. Application of 3D Bioprinting in Spinal Cord Injury Repair

As discussed in the previous section, the main aspects of SCI cure are the regeneration of functional neurons at the injured area with an adequate connection to the neighborhood tissues and, thus, the restoration of the nerve conduction function [51]. It was initially suggested that NSCs should be directly implanted in the injured area, as this may lead to the production of tissue with partial neural circuits and functions; however, it may also show limitations, such as uncontrolled differentiation of NSCs and a high number of dead implanted cells [52]. In this case, applying tissue engineering methods could help generate replaceable neural tissue with the required functionality. Generally, the physical support for regenerated tissue is provided by 3D scaffolds, which should have some specific properties if they are to be used inside the body. These properties include but are not limited to the following [53]:

- They should be made of biocompatible materials to improve the attachment and proliferation of cells and to guarantee the lack of immune and cytotoxic reactions. Moreover, these materials should also be biodegradable to ensure the substitution of the scaffold with the regenerated tissue in a specified time.
- They should have enough mechanical strength to ensure a low-stress level in the lesion region and to prevent collapse in this area throughout regular motion.
- They should contain an interconnected pore size at the microscale level to mimic the extracellular matrix of the natural tissue and to facilitate waste and nutrient exchange.
- Finally, they should have electrical conductivity to assist in neurite growth and neuro-regeneration.

The conventional methods used for the production of scaffolds are gas foaming, melt molding, electrospinning, sacrificial templating, and phase separation [54,55]. Gas foaming a low-cost method used to produce a porous 3D structure in which polymers are used in combination with a gas foaming agent that acts as a plasticizer and eliminates the glass transition and/or melting temperature of the polymers. It is a clean and flexible method for the production of porous structure at the micron scale; however, it also has some limitations restricting its wide application. For instance, it cannot be used for crystalline polymers since it cannot decrease the glass transition temperature of these polymers [56].

In the melt molding technique, a mixture of porogen and polymeric powder is prepared inside the mold and heated under pressure above the polymer glass transition temperature. This heating process leads to the production of a scaffold by making connections between polymer and porogens. Then, the porogens are extracted by immersing the mold in water, and, subsequently, the porous structure is fabricated. Although utilizing this method could produce a porous structure with a controllable shape, the high probability of contamination in the pores could restrict the application of this method [57].

Electrospinning is another interesting method, and it has been widely used in recent research. This technique is based on applying high-voltage electricity to a polymeric solution to fabricate fibrous scaffolds. A suitable scaffold could be prepared by controlling different parameters of the spinning process, thereby providing an appropriate platform for tissue engineering applications. That is, it could present a nanoscale/microscale porous structure that could mimic the natural extracellular matrix of tissues. Moreover, it could contain bioactive factors and functional drugs needed to regenerate the function of a tissue. For instance, this technique could be used in nerve tissue regeneration via the formation of nerve guidance conduits (NGCs), which are suitable for the self-repairing process. The main limitation of this method is the utilization of solvents that could be toxic to live cells. Moreover, the dense fibrous structure of the electrospun scaffold could limit their applications in 3D tissue engineering due to poor cell infiltration [1,58].

As is well known, most of the techniques mentioned above encounter limitations that could diminish their usage in tissue engineering applications. In recent years, a new powerful technique known as additive manufacturing (AM) has been introduced by scientists for the fabrication of scaffolds and it is widely used in different fields, from aerospace, automobile manufacture, and construction to different areas of medical science, especially tissue engineering. It is based on the fabrication of a 3D structure during a layer-by-layer fabrication process with the help of computer-aided design (CAD) or computer tomography (CT) scan images. This technique was introduced in 1986 by Charles W. Hull, who used stereolithography (SLA) to form 3D systems based on using a preprogrammed computer-controlled moving laser beam. Using AM in tissue engineering could overcome most of the limitations of other techniques via control of the porosity, chemistry, and complexity of the fabricated scaffold [59].

Different types of natural and synthetic polymers, ceramics, and metals could be used to fabricate 3D printed scaffolds. In addition to SLA, several other types of printing methods have been introduced, such as multi-jet modeling (MJM), selective laser sintering (SLS), digital light processing (DLP), laminated object manufacturing (LOM), fused deposition modeling (FDM), and micro-extrusion. SLA and DLP are based on utilizing liquid photopolymer resins and ultraviolet (UV) lasers. MJM is a type of inkjet bioprinting process that is based on the use of print head technologies for layer-by-layer deposition of photo-curable plastic resins. FDM is a simple method that uses thermoplastic filaments for printing; SLS uses a high-power laser to fuse small particles to fabricate 3D structures. LOM is based on the use of sheet materials in the form of stacking layers, and, finally, micro-extrusion is based on utilizing micro-size nozzles for the synthesis of miniaturized scaffolds with enhanced accuracy [60,61].

Among the various types of AM methods, inject printing, SLA, fused deposition modeling (FDM), and micro-extrusion are the most common and extensively used methods for the production of 3D printed scaffolds [62]. The advantages and disadvantages of these methods are presented in Table 2.

**Figure 3:**
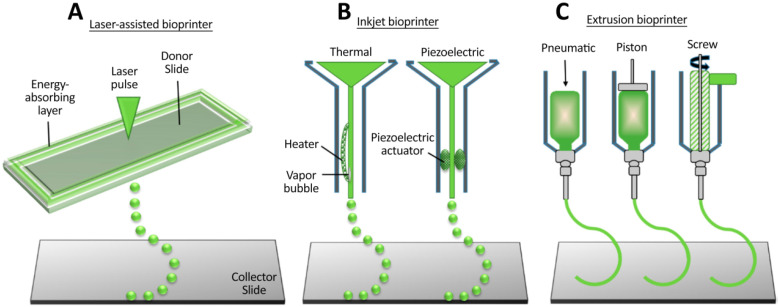
The most prevalent 3D bioprinting methods, which are used for tissue engineering applications. (**A**) Laser-assisted bioprinting, (**B**) Inkjet bioprinting, and (**C**) Microextrusion [64].

As mentioned in the above section, one of the most important areas where the 3D printing technique could be applied is the fabrication of tissue constructs for regenerative medicine. This type of 3D printing is known as 3D bioprinting, and it is conceptually similar to 3D printing. Generally, in 3D printing, materials such as polymeric resins, metal, plastic, and rubber are used to print the desired structure, while in 3D bioprinting, biological materials or bioinks are employed. Bioinks are typically laden with living human or mammalian cells and usually have growth factors or biomaterial that could increase the bioactivity of scaffolds. Among the mentioned methods, microextrusion, laser-assisted bioprinting, and inkjet bioprinting are suitable for bioprinting applications (Figure 3). In the extrusion method (or robotic method), biopolymers or cell-laden hydrogels are dispensed through a nozzle using mechanical systems or air pressure. In laser-assisted bioprinting, laser pulses are applied to create high pressure on the donor slide, directing the cell-laden hydrogel droplets to the collector part. In inkjet printing, cell-laden hydrogels or biopolymer droplets are ejected via a nozzle by utilizing a piezoelectric actuator or thermal energy [64].

Before the printing process, one must consider the important features of the bioink (i.e., viscosity, crosslinking, and gelation properties) that can affect the quality and morphology of the scaffold and its ability in adsorbing cells, which, in turn, can promote their proliferation. Moreover, the most probable limitations of using bioprinting techniques for regenerative medicine applications are legal, ethical, and social concerns, and, as such, these must be addressed before their utilization in clinical applications [65].

SCI contusions lead to an irregularly shaped cavity in the injury site, which is surrounded by preserved white matter. The ideal way of reducing further damage to the injury site is the insertion of biomaterials in the cavity to improve the treatment process. Bioprinted scaffolds are among the most interesting candidates that could be used as filler in the injury site. They can mimic the structure of the extracellular matrix and promote cell proliferation and tissue regeneration, as well as being able to improve the regrowth of axons in the lesion and restoring neural circuitry [66].

One of the most interesting features of 3D printed scaffolds, which distinguishes them from other type of scaffolds for SCI applications, is the addition of cells in their structure during the synthesis process. This allows for a dense and uniformly distributed structure to be prepared, as well as providing a good microenvironment for cell–cell and cell–scaffold interactions in the lesion region, thereby facilitating neuron regeneration and reinstate neural circuitry [66]. The following section summarizes some of the recent studies in which 3D bioprinted scaffolds were used in combination with different types of stem cells for SCI therapy applications.

### Combination of 3D Bioprinted Scaffolds and Stem Cells for SCI Therapy

To date, various studies with the aim of identifying a suitable method for the treatment of spinal cord injuries have been conducted. Among such methods, the simultaneous application of 3D bioprinting and stem cells has become the subject of considerable interest. Several types of hydrogels and biomaterials have been used for the fabrication of scaffolds for SCI research [67]. The constructed scaffold should have features that can provide specific physicochemical and biological conditions for stem cell attachment and promote their differentiation into neural phenotypes. In addition, bioprinting could result in acceptable models with favorable biochemical and mechanical properties and tunable microstructures for the evaluation of SCI. By controlling different synthetic parameters, such as shear stress, temperature, and pressure, scientists can produce scaffolds with a high number of live cells. Bioink is another important factor, and it should be considered during the fabrication of scaffolds with stem cells, as it could directly affect the viability of the cells and their differentiation ability [68,69].

By considering all of the above-mentioned features, Vega and coworkers fabricated a 3D bioprinted scaffold utilizing fibrin-based bioink and human-induced pluripotent stem cells (hiPSCs) to study SCI. They provided structures with more than 81% viable cells, in which the stem cells could be differentiated into motor neurons while expressing the neuronal marker 15 days after culturing [70].

**Figure 4:**
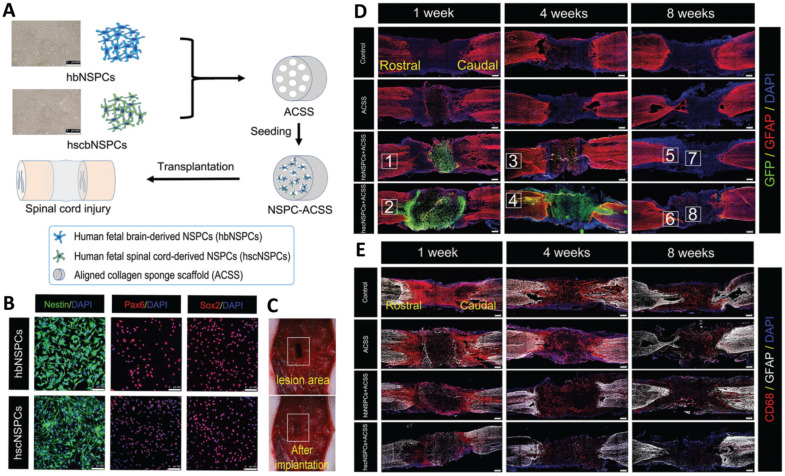
(**A**) Schematic of transplantation of hbNSPCs and hscNSPCs into the aligned collagen scaffold, Scale bars: 200 μm. (**B**) Immunofluorescence images of hbNSPCs and hscNSPCs, Scale bars: 100 μm. (**C**) Transplantation of the scaffold in an animal model. (**D**) Survival images of transplanted cells (hbNSPCs and hscNSPCs) after 1, 4, and 8 weeks. The images clearly show that hbNSPCs cells are not alive after 4 weeks (green dye) (the red dye responded to astrocytes), Scale bars: 250 μm. (**E**) Effect of two types of stem cells on suppression of inflammatory agents. hscNSPCs showed a better anti-inflammatory effect than that of hbNSPCs, Scale bars: 250 μm. Reprinted from [2] with permission from the Royal Society of Chemistry.

An aligned collagen scaffold was also used with neural stem/progenitor cells (NSPCs) for SCI therapy. It was revealed that NSPCs can fabricate a conducive microenvironment for neuronal regeneration and recovery of functions. The fabricated scaffold could provide support for cellular growth and differentiation and a guide for axonal extension. Moreover, it could promote neuro-regeneration and remyelination. The effect of stem cell source on SCI therapy performance has also been examined via the use of two types of NSPCs (brain-derived NSPCs (hbNSPCs) and spinal cord-derived NSPCs (hscNSPCs). The results revealed that spinal-derived stem cells acted more effectively than brain-derived NSPCs in promoting cell survival and neuronal differentiation and reducing the formation of glial scar and inflammatory agents while also improving the recovery of the locomotor functions in tested animal models (Figure 4) [2].

The combined use of iPSC-derived neural stem cells (iPSC-NSCs) and activated Schwann cells (ASCs) loaded on polycaprolactone (PCL) scaffolds was examined on an SCI animal model. The results of this study confirmed the cells’ effectiveness in reducing lesion cavity volume and improving the recovery of locomotor. Utilizing ASCs could improve neuronal survival and induce axon myelination. The co-culturing of ASCs by stem cells could accelerate neuron differentiation and enhance nerve ending recovery. In addition, the PCL scaffold has excellent properties, such as mechanical stability, biodegradability, biocompatibility, and high processability, which make it appropriate for cell transplantation [71].

To increase the scaffolds’ physicomechanical performances, it is possible to use composite hydrogels instead of simple ones, thereby benefiting from the advantages of different materials and thus meeting the mechanical and physiological requirements of the host tissues [72]. For instance, the composite of collagen/heparin sulfate scaffolds has been produced for SCI application with uniform pore distributions and improved mechanical and neurological properties. This composite could exhibit high biocompatibility in the presence of stem cells and also acted as a good carrier for basic fibroblast growth factor (bFGF) immobilization [73].

In a recent study, the combination of NSCs and a 3D bioprinted collagen/silk fibroin scaffold promoted nerve regeneration after SCI in rat models. The results of this study showed a significant effect of the combined use of both factors compared to that of sole factors. Indeed, the combined use of scaffold and stem cells could abundantly regenerate axons, reduce glial scarring, and elevate the amounts of neurological scores [74].

**Figure 5:**
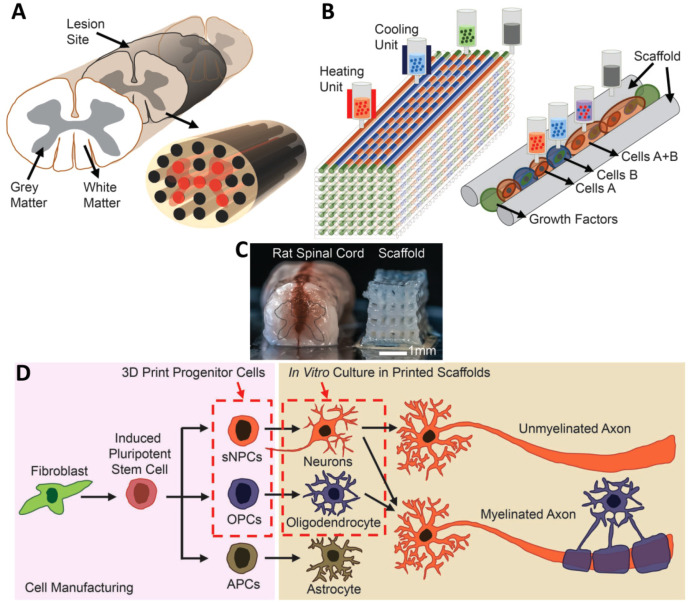
(**A**) Schematic of the spinal cord and 3D bioprinted scaffold. (**B**) Schematic of the extrusion bioprinting process. (**C**) Comparison of a real spinal cord and the fabricated scaffold. (**D**) Schematic of differentiation of iPSCs into three different types of neuronal cells. Reprinted from [75] with permission from Wiley Online Library.

In another study, the extrusion method was used for the fabrication of a multi-material 3D bioprinting scaffold containing clusters of iPSC-derived spinal neural progenitor cells (sNPCs) and oligodendrocyte progenitor cells (OPCs) (Figure 5). The NPCs could differentiate, and axons were extended throughout the scaffold. The combination use of stem cells and OPCs improved the recovery of axonal connections across the injured areas [75].

**Figure 6:**
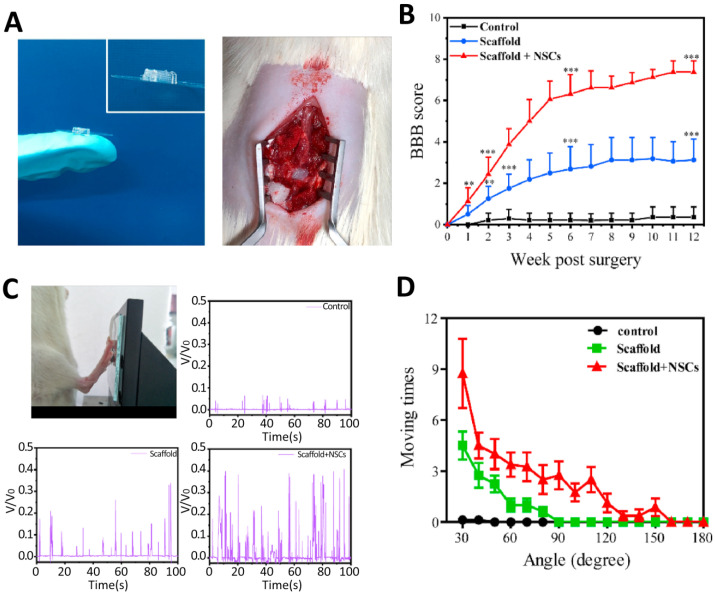
Functional recovery of SCI rats after three months of implantation. (**A**) Photographs of the scaffold with parallel linear arrays and implantation of the 3D bioprinted scaffold into the gap of the lesion area of SCI rats. (**B**) Basso-Beattie-Bresnahan (BBB) scores of rats in each group, ** *p* < 0.01, *** *p* < 0.001. (**C**) Image of SCI rats kicking the pressure sensor. Record of hindlimbs of SCI rats kicking the pressure sensor, and their relative voltages corresponding the hindlimb movement of SCI rats in the control, scaffold, and scaffold + NSCs groups. (**D**) Ankle angle distribution of SCI rats in the control, scaffold, and scaffold + NSCs groups). Reprinted from [66] with permission from Elsevier.

Liu et al. [66] used microextrusion to fabricate 3D scaffolds for SCI repair (Figure 6). Their study used a cell-laden bioink consisting of chitosan, hyaluronic acid derivatives, Matrigel, and NSCs. This bioink showed fast gelation (within 20 s) and spontaneous covalent crosslinking capability, facilitating convenient one-step bioprinting of spinal cord-like constructs. These authors also investigated the performance of scaffolds using an in vivo rat model. Their results showed that the printed scaffolds have high cell viability, i.e., 95%, and could improve neural tissue regeneration while decreasing glial scar deposition. In Table 3, a summary of some recent works in this area is presented.

## 4. Conclusions and Future Perspectives

Neural tissue engineering is an interesting field of science that could facilitate function recovery at the injured site of the nervous system via the use of therapeutic agents. To date, much effort has been made in the field of SCI treatment, and several types of therapeutic methods have been introduced, among which the most interesting are 3D bioprinting scaffolds, which are capable of combining neural grafts with different bioactive factors and cells. The 3D bioprinting technique is a rapidly growing method that could be used in the generation of highly complex scaffolds with the ability of holding stem cells, which are good candidates for the promotion of regeneration of neurons and accelerating the treatment process. Three dimensionally bioprinted scaffolds, which are fabricated via different methods, could overcome the limitations of other methods, such as their high cost, complexity, and risk of immune rejection and activation. On the other hand, they may also have limitations in terms of the types and number of used materials, the resolution of the constructed bioprint, and the maintenance of the viability of stem cells and promotion of their differentiation. Moreover, several aspects must be addressed before a cell type can be distributed as a commercially viable cell source, including safety, efficiency, cost, and the possibility of large-scale manufacturing. Each stem cell type has advantages and disadvantages, and it is still needed to determine which cell type shows the most effective treatment for SCI. Information obtained from stem cell-based treatment studies revealed that the use of stem cells could be a promising alternative for SCI therapy. However, numerous studies are needed to find the optimal bioinks and methods for the combined use of 3D bioprinted scaffolds and stem cells in clinical trials. Moreover, 4D printing, which is a new, effective method of fabricating smart responsive scaffolds that can enhance the dynamic interactions between cells and scaffolds, has also been developed; however, this technique is also in its infancy and warrants further investigation.

## Figures and Tables

**Table 1:** The advantages and limitations of different types of stem cells in SCI therapy.

Type of Stem Cells	Benefits	Restrictions	Ref.
Embryonic stem cells	Remarkable proliferative capacity In vitro and in vivo pluripotency Ability to differentiate into cells of ectodermal origin Promotion of the sprouting function Providing a scalable, tractable, and accessible high-throughput platform for decoding mammalian embryogenesis at a high level of resolution İnducing myelination Good source of differentiation to oligodendrocytes and motoneurons	Ethical controversies Partial recovery of forelimb motor after being transplanted into both chronic and acute cervical spinal cord injury rat models Probability of tumor formation	[35,36,47]
Induced pluripotent stem cells	Prevention of ethical considerations and immunological rejection via use of patient-specific iPSCs Inhibition of neuronal apoptosis Promotion of myelin production by oligodendrocytes Modulation of immunopathological events	Inhibition of restorative effects of the transplantation via GAS5 silencing gene Several risks, such as uncontrolled expression of integrated transgenes, insertional mutagenesis, tumor formation, and silencing or downregulation of transgenes Aberrant reprogramming Presence of transgenes	[39,40,47]
Mesenchymal stem cells	Easy isolation (from different sources) Good preservation Reduction in ethical concern Reduced risk of tumors development High regenerative potential after freezing Rapid proliferation Obtaining high multilineage differentiation Low immunoreactivity “Homing” capability Control of the effects of secondary injury (after SCI) Establishment of higher recovery of nerve functions	Low survival rate Lack of enough evidence on differentiating MScs to neuronal cells Differentiation of transplanted cells into other types of cells, such as osteoblasts, which limits their therapeutic effects Unsatisfactory translation from small animal experimental models (mice and rats) into human clinical practice Usually have paracrine activity instead of cellular replacement mechanisms	[48]
Neural stem cells	Promotion of remyelination of axons High self-renewal capability in in vitro culturing More preferable than hESCs in clinical applications Less potential of tumor formation	Glial scar formation Limited differentiating potential in clinical trial after several passages Their cell survival and integration highly dependent on their source of transplantation and isolation methods Need purification Moderate cell survival İnefficient tracking systems Lack of neurotrophic factors	[49]

**Table 2:** Pros and cons of the AM methods used for tissue engineering applications [63].

Different Types of AM Methods	Advantages	Disadvantages
Inkjet bioprinting	Cost-effective technique High-speed printing and good resolution Cell printing ability	Produces structures with low mechanical properties High cell concentrations (>5 million cells/mL) must be seeded Possible harm to cells due to heating ink during the printing process
Fused deposition modeling (FDM)	Simple process Low cost High-speed printing	Due to the high temperature at the nozzle, it is not suitable for cell printing Poor surface property
Stereolithography (SLA)	Very high printing resolution High surface quality	Slow and costly Restriction of types of materials for 3D printing is possible
Micro-extrusion	Cost-effective method High viscosity ink can be printed Ink with high cell density can be used High-speed printing	Shear stress at the nozzle may cause harm to cells

**Table 3:** Some previous studies related to 3D bioprinting applications of neural tissue engineering.

Print Method	Materials and Cell	Type of Study	Results	Ref.
Microextrusion	Polyurethane-PCL-NSCs	In vitro	High cell growth and differentiation were observed on the scaffold.	[76]
Microextrusion	Composite hydrogel of alginate, carboxymethyl chitosan, and agarose laden with NSCs	In vitro	Cell viability and differentiation were observed.	[77]
SLA	GelMa, graphene nanoplatelet-NSCs	In vitro	Homogenous cell distribution throughout all scaffolds was observed, and neurites spread from soma after 14 days of culture.	[78]
SLA	Composite hydrogel GelMa and PEGDA-NSCs	In vitro	Light stimulation increased NSC neuronal differentiation and inhibited the generation of glial cells.	[79]
SLA	Poly(3,4-ethylenedioxythiophene) (PEDOT): polystyrene sulfonate (PSS)-dorsal root ganglia (DRG) cells	In vitro	Conductive hydrogel improved regulation and stimulation of cell behavior.	[80]
Microextrusion	Collagen-heparin sulfate- NSCs	In vivo (rat SCI model)	Improved locomotor function was observed.	[73]
Microextrusion	Gelatin/fibrin and GelMa-neural progenitor cells (NPCs)	In vitro	Bioprinted NPCs differentiated and extended axons throughout microscale scaffold channels.	[75]
Micro-scale continuous projection printing (μCPP)	PEGDA-GelMa-NPCs	In vivo (rat SCI model)	Injured host axons were regenerated in the scaffolds and formed synapse onto NPCs implanted into the scaffold.	[81]
Microextrusion	chitosan, hyaluronic acid derivatives, and Matrigel-NSCs	In vivo (rat SCI model)	Bioprinted scaffolds promoted axon regeneration and decreased glial scar deposition, leading to significant locomotor recovery of SCI model rats.	[66]

## Data Availability

Not applicable.

## Abbreviations

AAV2	Adeno-associated virus
AM	Additive manufacturing
ASCs	Activated Schwann cells
bFGF	Basic fibroblast growth factor
Bcl-2	B-cell lymphoma-2
BDNF	Brain-derived neurotrophic factor
BBB	Basso–Beattie–Bresnahan
CCL2	Chemokine (C-C motif) ligand 2
CCL5	Chemokine (C-C motif) ligand 5
CNS	Central nervous system
CAD	Computer-aided design
CT	Computer tomography
DLP	Digital light processing
DRG	Dorsal root ganglia
EGF	Epidermal growth factor
FDM	Fused deposition modeling
FGF	Fibroblast growth factor
Gap-43	Growth-associated protein 43
GAS5	Growth arrest-specific 5
GDNF	Glial cell-derived neurotrophic factor
GelMA	Gelatin methacryloyl
GFAP	Glial fibrillary acidic protein
hbNSPCs	Brain-derived NSPCs
hESC-NS	Human embryonic stem cell-derived neural stem cells
hiPSCs	Human-induced pluripotent stem cells
hNSCs	Human neural stem cells
HRE	Hypoxia response element
hscNSPCs	Spinal cord-derived NSPCs
IL-4	Interleukin 4
IL-13	Interleukin 13
iPSCs	Induced pluripotent stem cells
iPSC-NSCs	iPSC-derived neural stem cells
lncRNA-GAS5	Long non-coding RNA-growth arrest-specific transcript 5
LOM	Laminated object manufacturing
MJM	Multi-jet modeling
MRI	Magnetic resonance imaging
NGC	Nerve guidance conduits
NSCs	Neural stem cells
NSPCs	Neural stem/progenitor cells
NPCs	Neural progenitor cells
NT-3	Neurotrophin-3
OPCs	Oligodendrocyte progenitor cells
PACAP	Pituitary adenylate cyclase-activating peptide
PCL	Polycaprolactone
PEDOT	Poly(3,4-ethylenedioxythiophene)
PEGDA-NSCs	Poly(ethylene glycol) diacrylate-neural stem cells
PSS	Polystyrene sulfonate
RT-PCR	Real-time polymerase chain reaction
SCI	Spinal cord injury
SEM	Standard error of mean
Siglec-9	Sialic acid-binding Ig-like lectin 9
SLA	Stereolithography
sNPCs	Spinal neural progenitor cells
SLS	Selective laser sintering
TrkB	Tropomyosin receptor kinase B
3D	Three-dimensional
UV	Ultraviolet
